# Acceptability and Feasibility of a Patient-Oriented Music Intervention to Reduce Pain in the Intensive Care Unit: Protocol for a Crossover Pilot Randomized Controlled Trial

**DOI:** 10.2196/40760

**Published:** 2023-05-10

**Authors:** Melissa Richard-Lalonde, Nancy Feeley, Sylvie Cossette, Linda L Chlan, Céline Gélinas

**Affiliations:** 1 Ingram School of Nursing McGill University Montreal, QC Canada; 2 Centre for Nursing Research Integrated University Health and Social Services Centre for West-Central Montreal Jewish General Hospital Montreal, QC Canada; 3 Faculté des Sciences Infirmières Université de Montréal Montreal, QC Canada; 4 Centre de Recherche Institut de Cardiologie de Montréal Montreal, QC Canada; 5 Division of Nursing Research Department of Nursing Mayo Clinic College of Medicine and Science Rochester, MN United States

**Keywords:** music, pain, intensive care unit, pilot, feasibility, acceptability

## Abstract

**Background:**

Many patients experience pain in the intensive care unit (ICU) despite receiving pain medication. Research has shown that music can help reduce pain. Music interventions studied so far have not used music streaming to generate playlists based on patient preferences while incorporating recommended tempo and duration. Previous research has focused on postoperative ICU patients able to self-report, which is underrepresentative of the ICU population that might benefit from a music intervention for pain management. We developed a new patient-oriented music intervention (POMI) that incorporates features based on theoretical, empirical, and experiential data intended to be used in the ICU. Such a music intervention should consider the expertise of ICU patients, family members, and nursing staff, as well as the practicality of the intervention when used in practice.

**Objective:**

The primary objectives of this study are to (1) evaluate the acceptability and feasibility of the POMI to reduce pain in ICU patients and (2) evaluate the feasibility of conducting a crossover pilot randomized controlled trial (RCT) for intervention testing in the ICU. A secondary objective is to examine the preliminary efficacy of the POMI to reduce pain in ICU patients.

**Methods:**

A single-blind 2×2 crossover pilot RCT will be conducted. Patients will undergo 1 sequence of 2 interventions: the POMI which delivers music based on patients’ preferences via headphones or music pillow for 20-30 minutes and the control intervention (headphones or pillow without music). The sequence of the interventions will be inverted with a 4-hour washout period. Timing of the interventions will be before a planned bed turning procedure. Each patient will undergo 1 session of music. Twenty-four patients will be recruited. Patients able to self-report (n=12), family members of patients unable to self-report (n=12), and nursing staff (n=12) involved in the bed turning procedure will be invited to complete a short questionnaire on the POMI acceptability. Data will be collected on the feasibility of the intervention delivery (ie, time spent creating a playlist, any issue related to headphones/pillow or music delivery, environmental noises, and intervention interruptions) and research methods (ie, number of patients screened, recruited, randomized, and included in the analysis). Pain scores will be obtained before and after intervention delivery.

**Results:**

Recruitment and data collection began in March 2022. As of July 5, 2022, in total, 22 patients, 12 family members, and 11 nurses were recruited.

**Conclusions:**

Methodological limitations and strengths are discussed. Study limitations include the lack of blinding for patients able to self-report. Strengths include collecting data from various sources, getting a comprehensive evaluation of the intervention, and using a crossover pilot RCT design, where participants act as their own control, thus reducing confounding factors.

**Trial Registration:**

ClinicalTrials.gov NCT05320224; https://clinicaltrials.gov/ct2/show/NCT05320224

**International Registered Report Identifier (IRRID):**

DERR1-10.2196/40760

## Introduction

### Background and Rationale

Pain is a common symptom in critically ill adults, both in patients able and unable to self-report [1]. Guidelines recommend the use of a multimodal approach to pain management to reduce opioid use and optimize pain relief [1]. Music has been suggested as a nonpharmacologic intervention in acute and chronic care settings, but little is known about its efficacy and feasibility in the intensive care unit (ICU) [2-8]. We conducted a systematic review and meta-analysis of randomized controlled trials (RCTs) to establish the efficacy of music in the adult ICU. Music interventions of 20 to 30 minutes were effective to reduce pain by almost 2 points on a 0-10 numeric rating scale (NRS) in ICU patients able to self-report [9]. However, the effect of music on pain in ICU patients unable to self-report remains unknown. In a previous review, some studies reported that family members of ICU patients expressed their interest in participating in the music selection process and in the pain management of their loved ones [10]. Therefore, music interventions for patients unable to self-report could involve the participation of family members based on their intimate knowledge of the patient and their music preferences.

Current recommendations for music interventions in postoperative patients are to provide music in the range of 60-80 beats per minute (bpm) [11]. However, most ICUs do not have access to music therapists, who have the expertise to provide personalized music within this range. Thus, there is a need to develop an easy-to-use music intervention that produces individualized music playlists with a tempo of 60-80 bpm for ICU patients. Music streaming services allow for the music selection of specific tempo ranges and should be explored as a simple means to provide a more accessible music intervention in the adult ICU.

Studies conducted thus far have mainly focused on postsurgical patients, mechanically ventilated, and able to communicate despite the fact that many patients are likely to be unable to communicate during their ICU stay [9,12,13]. Therefore, RCTs conducted until now on the effect of music to reduce pain in critically ill adults have limited generalizability to the entirety of the ICU population, despite the knowledge that all patients can experience pain and could benefit from this nonpharmacological pain management intervention.

Another limitation in previous RCTs analyzed in the systematic review relates to sample size [9]. In 4 RCTs, no sample size calculation was reported [14-17]. In 3 RCTs, the sample size was calculated based on outcomes other than pain [18-20]. In another 3 studies, the calculated sample size required was not attained for various feasibility issues attributed to “slow” or “difficult” recruitment (due to time limit, refusal rate due to randomization or family visits, and narrow inclusion criteria), or withdrawal of ICU adult patient participants who did not like the music chosen for them [21-23]. In 1 study, it was unclear whether the sample size represented the number of ICU adult patient participants or the number of observations [24]. Inadequate power may explain why 11 of the 18 RCTs found a significant pain reduction while 7 did not. Therefore, there is a need to evaluate the feasibility of research methods by conducting a pilot RCT prior to evaluating the efficacy of any new music intervention in the adult ICU.

### Objectives

This study aims to (1) evaluate the acceptability and feasibility of a new patient-oriented music intervention (POMI) to reduce pain in ICU patients (primary objective); (2) evaluate the feasibility of conducting a crossover pilot RCT for intervention testing in the adult ICU (primary objective); and (3) examine the preliminary efficacy of the POMI (secondary objective).

### Trial Design

A single-blind 2×2 crossover pilot RCT was selected for this study protocol, where patients undergo a sequence of 2 intervention periods: the POMI and the control intervention (CTL: headphones or pillow without music), with an allocation ratio of 1:1.

## Methods

### Design

**Figure 1:**
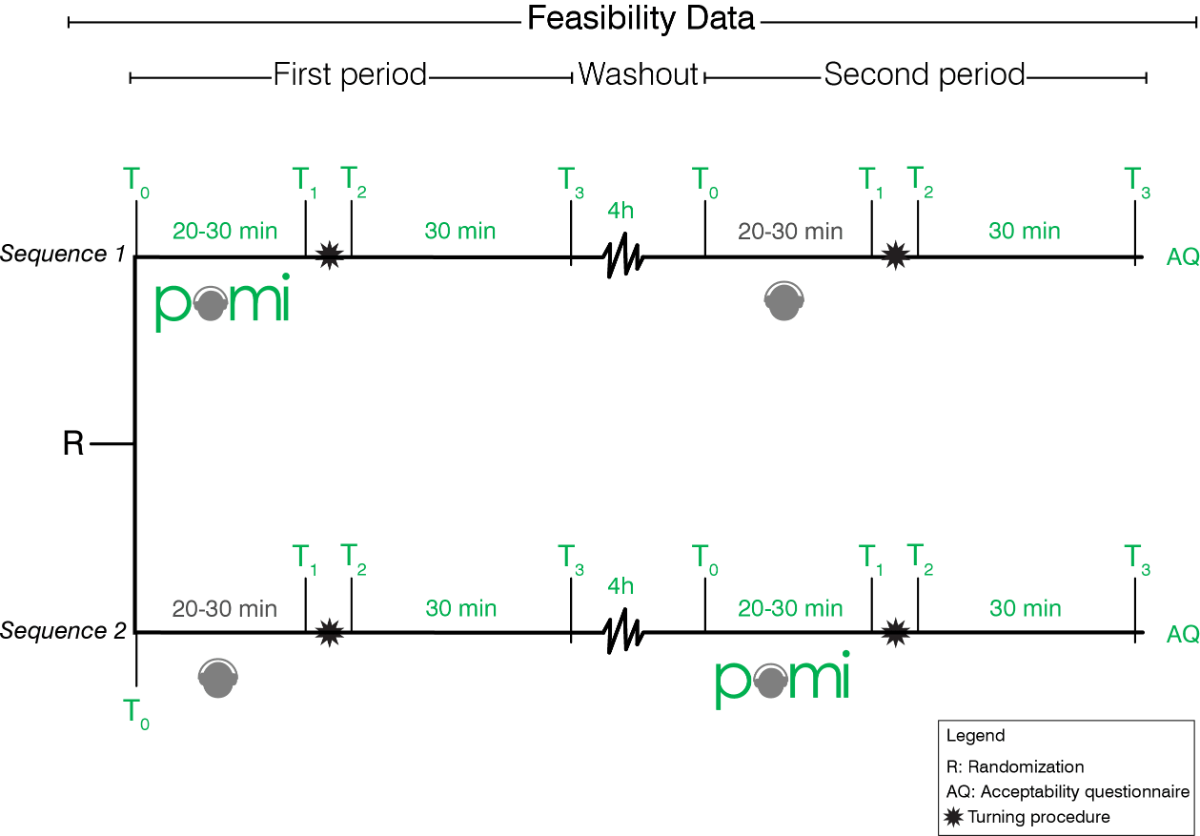
Study design for the 2×2 crossover pilot randomized control trial.

A single-blind 2×2 crossover pilot RCT is being conducted to evaluate the acceptability, feasibility, and preliminary efficacy of the POMI. Recruitment is planned to occur over a period of 6 months during which we aim to enroll a total of 24 patient participants. As shown in Figure 1, each participating patient is randomly assigned to 1 sequence (sequence 1 or 2) of 2 intervention periods: the POMI and the CTL (headphones or pillow without music). Patients in sequence 1 receive the POMI during the first intervention period, followed by the CTL in the second intervention period; and patients in sequence 2 receive the CTL first, followed by the POMI. Each 20-30–minute intervention period is provided before a bed turning procedure that is planned as part of the participating patient’s usual care by the nursing staff. There is a 4-hour minimum washout period between both intervention periods, with data collected during the day and evening. Data collection begins as soon as possible following recruitment, always in coordination with patient care. Therefore, the first period of data collection may occur in the daytime or in the evening.

**Figure 2:**
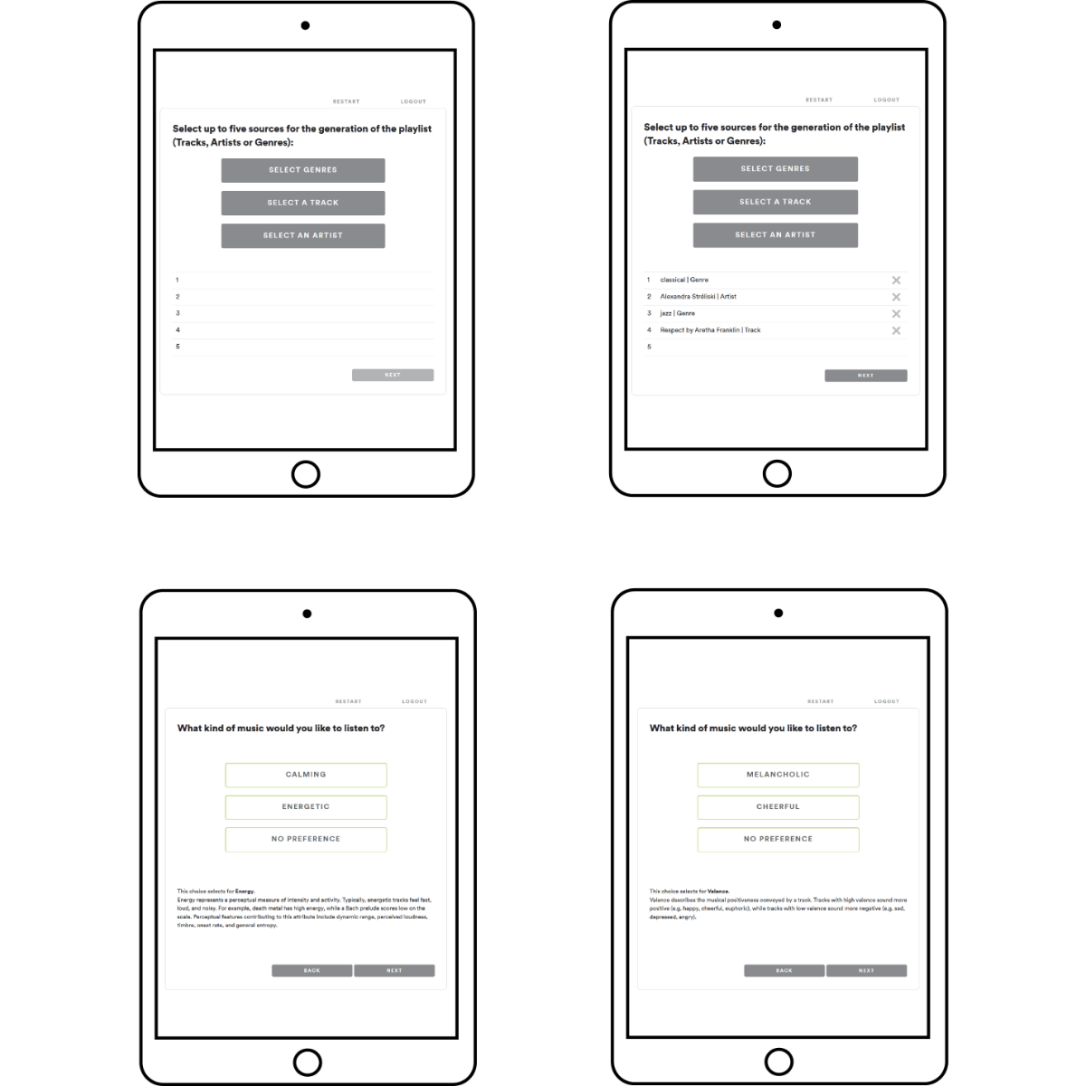
Example of web-based tool screenshots for the patient-oriented music intervention.

### Recruitment

Recruitment is conducted by the first author (MRL), who is introduced to the eligible patient and family member by the nurse caring for the eligible patient. If the eligible candidate is interested in hearing about the study, the student researcher meets with them at the ICU bedside to present the study, provides a copy of the informed consent form, and answers any question. The eligible candidate is then given time to think about whether they are interested in the study. ICU nursing staff (ie, nurses and orderlies) involved in the turning procedure during the study are invited individually prior to the scheduled bed turning procedure. Eligible nursing staff are given the informed consent form and can choose to participate at their convenience. Eligible criteria are detailed in Table 1.

**Table 1:** Eligibility criteria.

			Type of participant						
			Patient able to self-report		Patient unable to self-report		Family member		Nursing staff
**Inclusion criteria**									
	Is ≥18 years old	✓		✓		✓		✓	
	Is admitted to ICU^a^	✓		✓					
	Has a loved one admitted to the ICU					✓			
	Works in the ICU							✓	
	Is able to self-report	✓				✓		✓	
	Is able to listen to music as per patient or a family member for patients unable to self-report	✓		✓					
	A family member is present at the bedside			✓					
	Considers self to have knowledge of the patient's music preferences					✓			
	Is qualified to consent to any care required by the state of health for the incapable ICU adult patient					✓			
	Is present during the turning procedure at the time of the POMI^b^ project data collection							✓	
**Exclusion criteria**									
	Cannot be turned	✓		✓					
	Does not speak or understand French or English	✓		✓		✓		✓	
	Is unarousable, as defined by a score of −5 on the Richmond Agitation Sedation Scale (RASS)	✓		✓					
	Is under the effects of neuromuscular blocking agent	✓		✓					

^a^ICU: intensive care unit.

^b^POMI: patient-oriented music intervention.

### Participants

#### Sample Size

A minimal sample size of 10 participants per group is recommended in pilot studies [25,26]. Because studies with repeated measures (such as crossover designs) require more time commitment from participants (ie, multiple measurements over time), an attrition rate of approximately 15% (n=2 per group) can be anticipated, which is consistent with what has been reported in previous studies conducted in the targeted population [27,28]. To account for this, the recruited sample size was estimated to be 12 participants per group.

#### Patients

A sample size of 24 patients is targeted, with 12 being able to self-report and 12 unable to self-report. Patients able to self-report will be asked about their music preferences, levels of pain intensity, distress, and acceptability of the POMI.

#### Family Members

Family members are defined by the patient or, in the case of those unable to self-report, by their surrogates. In such cases, the family may be related or unrelated to the patient. Family members are the individuals who provide support and with whom the patient has a significant relationship [29]. A sample size of 12 family members is targeted: 1 for each patient unable to self-report. Family members will be responsible for providing information on the music preferences of their loved one unable to self-report, as well as answering questions on the POMI acceptability.

#### Nursing Staff

A total of 12 members of nursing staff involved in a participating patient’s bed turning procedure will be recruited to answer questions on the acceptability of the POMI from their perspective.

### Randomization

For the equal allocation of both groups, 24 opaque envelopes were prepared in advance by an independent member of the research team, with the use of a digitally generated list [30]. Once patients or their representatives consent to participate and agree to randomization, patient participants are categorized as either able to self-report or unable to self-report, producing 2 strata. Within each stratum, patients are randomized to either sequence 1 or sequence 2, following a permuted block randomization to ensure balance within each stratum. A block size of 4 was used (3 blocks per stratum) for a sample size of 24 ICU adult patient participants (in each block, 2 patient participants will be assigned to sequence 1 and 2 patient participants will be assigned to sequence 2, and the ordering will be random).

### Patient-Oriented Music Intervention

#### Overview

The brief name given to this intervention is POMI (patient-oriented music intervention). In POMI, music is delivered to adult patients either via headphones (Bose, QuietComfort 35) or by a music pillow (MusiCure, hospital grade). Adult patients admitted to the ICU and able to self-report can choose the mode of delivery based on their personal preference. Adult patients admitted to the ICU and unable to self-report are given the music pillow. For patients able to self-report, individualized music playlists are created based on the patient’s music preferences. For patients unable to self-report, a family member is asked about the patient’s music preferences. Questions about music preferences include music genre, track title, artist name, instrumentalness, acousticness, energy, and valence, as defined by the streaming service Spotify.

#### Playlist Creation

The personalized music playlist is generated prior to the POMI period for each patient participant. To determine the participant’s music preferences, the following questions are asked, based on possible recommendations through the Spotify Application Programming Interface [31]:

1. Is there any music genre that you would like to listen to?
2. Is there any specific song title that you would like to listen to?
3. Is there any music artist that you would like to listen to?
4. Would you like to hear music that is more instrumental, more vocal, or do you have no preference?
5. Would you like to hear music that is more acoustic, more electric, or do you have no preference?
6. Would you like to hear music that is more calming, more energetic, or do you have no preference?
7. Would you like to hear music that is more cheerful, more melancholic, or do you have no preference?
8. Would you like to hear music that is more popular, less popular, or do you have no preference?
9. Would you like to hear music that is recorded in studio, do you prefer live recordings of music, or do you have no preference?

Any or all the questions can be skipped as preferred if at least 1 answer is given to questions 1, 2, or 3. Definitions of any of the music attributes (eg, electric and energetic) are provided if necessary and are available on the web-based tool.

The reported preferences are entered into a POMI web-based tool (Figure 2) to generate a personalized music playlist on Spotify, with a tempo restriction of 60-80 bpm as recommended [11]. Music is then played for 20 to 30 minutes via a smart device (iPad, 8th generation).

At all times, patients can control the music (eg, skip a song or stop the music, either briefly or permanently) by accessing the iPad themselves or communicating with the student researcher. Patients unable to self-report are continually monitored by the student researcher while the music is playing so that any nonverbal reaction to the music, indicating such dislike of the music, would lead the student researcher to stop the music immediately.

### Control Intervention

The CTL consists of providing a 20-30–minute period without music either while wearing headphones (Bose, QuietComfort 35) or with the head resting on a music pillow (MusiCure, hospital-grade), which is consistent with the mode of delivery for the POMI. Adult patients admitted to the ICU and able to self-report can choose 1 of the 2 modes of delivery. Patients unable to self-report are given the music pillow.

### Washout Period

To allow for any pain-reducing effect of the POMI to dissipate before the CTL, a washout period of at least 4 hours is scheduled between both intervention periods, based on previous data on the duration of the analgesic effect of music on pain [32].

### Video Recordings

Video will be recorded for the duration of the intervention period and at each pain assessment time point. These video recordings will be used to have an independent member of the research team evaluate: (1) the Critical-Care Pain Observation Tool (CPOT) scores for each patient participant (to ensure blinding of the research team member interrater to sequence allocation) and (2) the intervention fidelity.

### Outcomes

#### Acceptability

The acceptability questionnaire is adapted from the treatment acceptability and preferences (TAP) validated measure [33]. The TAP is comprised of 4 items: suitability, appropriateness, effectiveness, and willingness to comply, each of which is rated on a 5-point scale ranging from 0 (not at all) to 4 (very much) with higher scores indicating greater acceptability [33]. The total scale score is then obtained by calculating the mean of all the items’ scores. The TAP was shown to have a Cronbach α coefficient greater than .75, supporting good internal consistency [34], and it has been used to evaluate a variety of interventions in different acute and postsurgical care settings [35-37]. The TAP can capture “the complex nature of [participants’] preferences” and yet being simple enough for use in the ICU setting [33]. As recommended [38], 1 item has been added to the questionnaire to determine the risks of side effects of the POMI, an additional important aspect in assessing the acceptability of the intervention.

#### Feasibility of Intervention

The items for the assessment of intervention feasibility include (1) time spent (in minutes) creating the individualized playlist; (2) the presence or absence of any issue with headphone or pillow use; (3) the presence or absence of any issue with music delivery; (4) the presence or absence of skipping one or more songs from the generated playlist; (5) the presence or absence of any environmental noise (eg, alarms and voices) during intervention delivery; (6) the presence or absence of any POMI interruptions; (7) whether the patient participant received the full duration of the POMI; (8) the dose (duration in minutes) of the music delivered; (9) the characteristics of the music delivered (eg, music genres).

Additionally, as part of the feasibility of the intervention, the fidelity of the intervention will be assessed based on specific criteria [39] and will include meeting with participants to discuss music preferences, producing a personalized playlist, and playing music once at least for 20 minutes. Any issues with the delivery of the POMI will also be recorded.

#### Feasibility of Research Methods

The items for the assessment of the feasibility of research methods, based on the CONSORT guidelines for pilot and feasibility RCTs [40,41], include the number of patients screened, number of eligible patients, number of participants recruited, number of participants randomized, and number of participants included in the analysis.

#### Preliminary Efficacy of POMI on Acute Pain

Pain will be assessed at 4 different timepoints for each intervention period: before the intervention, immediately after the intervention, during the bed turning procedure, and 30 minutes after the bed turning procedure (Figure 1, T_0_-T_3_). Pain assessments will be performed using validated tools as recommended in ICU clinical practice guidelines [1]. For all patient participants, the CPOT will be used because it is one of the most valid behavioral scales for assessing pain in critically ill adults [42]. In addition to the CPOT, patient participants able to self-report will be asked to rate their pain intensity using the 0-10 Faces Pain Thermometer [43] and their pain distress on a 0-10 NRS [44,45].

### Data Analysis

#### Overview

A data bank will be created with the SPSS software (version 27.0; IBM Corp) [46], where the collected data on acceptability, feasibility, and preliminary efficacy will be entered. All the statistical analyses described below will be performed using SPSS.

#### Acceptability of the Intervention

The acceptability of the POMI will be determined using the TAP questionnaire. The frequencies, medians, and IQRs will be calculated for each item as well as for the total score, which will be calculated by taking the median, out of 4, of all items. The first 4 items (suitability, appropriateness, perceived effectiveness, and willingness to comply) will be scored in sequence (with 0 being the least favorable and 4 being the most favorable), whereas the last item (risks or side effects) will be scored in reverse (with 4 being the least favorable and 0 being the most favorable), as it is a negatively worded question. Any notes or comments added to the ratings will be compiled by category and presented descriptively to accompany the numerical ratings. A median above 2 out of 4 for the total score will be considered as an acceptable intervention, overall. An item median score above 2 out of 4 will be considered an acceptable attribute of the POMI. A median below 2 out of 4 will indicate the need to look more closely at the comments accompanying the ratings and modify the intervention to improve the acceptability of the POMI (eg, mode of delivery and dose). The acceptability of the POMI will be established via data triangulation from all study participants: patients, family members, and nursing staff [47].

#### Feasibility and Fidelity of Intervention

Descriptive statistics will be obtained to compute the frequencies for each of the intervention feasibility items. The POMI will be considered a feasible intervention if there are no issues in over 50% of the items for the assessment of the intervention feasibility (as listed above) for at least 80% of the patient participants in each group [48]. Regarding the fidelity of the intervention, descriptive data will be computed on the amount of time spent creating the music playlists (≤10 minutes), delivery of the overall POMI (use of headphones or pillow), as well as the amount of time the music will be listened to (once, for at least 20 minutes). The percentage of items completed on the fidelity checklist will also be computed in order to ensure that at least 80% of the intervention fidelity items will be delivered as planned, yet to allow for a certain amount of flexibility, if needed [48,49].

#### Feasibility of Research Methods

Descriptive statistics will be generated for each of the feasibility of research methods items. The screening and recruitment procedures will be described and include the number of patients screened, the proportion of eligible patients as well as the number of enrolled participants. If less than 50% of the potential patient participants are found to be eligible, considerations will be made to broaden the inclusion criteria in an eventual full-scale RCT [39]. Time to recruit will be considered adequate if 24 patient participants are enrolled within 6 months. All issues related to recruitment will be described and grouped into categories by the student researcher.

The retention rates will be calculated and expected to be above 80%. Reasons for participant withdrawal will be described, when known, and if retention rates are below 80%, strategies will be recommended to reduce attrition, based on the reasons for study withdrawal. If more than 10% of the participants (in each group) are found to have missing data, reasons for missing data will be described to inform how to reduce the amount of missing data in future research.

#### Preliminary Efficacy

Descriptive statistics will be computed for all outcomes (CPOT, pain intensity, and pain distress scores). The 95% CIs will be computed for each dependent variable at each time point.

Considering the small sample size for a pilot study, the potential efficacy of the POMI will be analyzed using nonparametric tests. For the dependent variables (CPOT, pain intensity, and pain distress scores), the Friedman test will be used to compare the scores at individual time points in each group separately. If the Friedman test is found to tend toward significant (*P*<.10), the Wilcoxon signed rank test will be used to compare 2 timepoints in pairs with a Bonferroni correction (.05 per number of tests) to locate the differences.

The preliminary efficacy findings will not be used to inform the decision to pursue a full-scale RCT. However, any tendency toward statistical significance (*P*<.10) or significant lower pain scores in the music period versus the control period will support that the intervention group might decrease pain over time, compared to the control group. A formal hypothesis of efficacy will need to be tested in a full-scale RCT, which will only be recommended if the POMI is deemed acceptable and feasible.

### Ethics Approval

Ethics approval was submitted in July 2021 and approved in December 2021 (Project #2022-3005). Participation in this research project is voluntary and ongoing for all participants. Participants are free to refuse to participate and may withdraw from this research study at any time, without having to give a reason, and without any consequence to them now or in the future. The participant’s decision not to participate in the study, or to withdraw from it, will have no impact on the quality of care and services to which they are otherwise entitled. Participants are free to refuse to answer any question and remain in the study. Participants are free to refuse to be video recorded.

For a patient who is unable to consent to participate in the study, a family member representative will provide the written consent on behalf of the patient. From this time and until the patient participant is discharged from the ICU, the student researcher will follow up on the ICU adult patients who were unable to consent to determine if they regain the ability to consent for themselves. In the case where a patient participant who was previously unable to consent regains the ability to consent at any time, before or after the intervention, the student researcher will present the research project and the information and consent form to allow the ICU adult patient to make an informed decision regarding their participation in the study.

The data collected from a participant as part of this study, excluding the video recordings, could be used for future research projects related to this study only with the participant’s explicit permission. The results of the research study, excluding video recordings, may be presented at conferences, published in specialized journals or be the subject of scientific discussions, or be used for teaching purposes. No identifying information will be published in any way.

At any time, participants have the right to consult their study file in order to verify the information gathered and to have it corrected if necessary. All study data will be stored safely for 10 years, after which time they will be permanently destroyed by being either shredded or permanently deleted from the server.

Patients and families will be offered the hyperlink to their playlist as well as a paper version of the list of songs played as part of the study. Nursing staff participants will be offered a US $20 gift certificate to compensate for their time spent participating in the study.

## Results

This study was registered to ClinicalTrials.gov (NCT05320224) in March 2022. Recruitment and data collection began in March 2022.

## Discussion

### Methodological Strengths

One strength of this study is the evaluation of the intervention acceptability from multiple sources: ICU patients, families, and nursing staff. This will allow us to gain access to the different perspectives of the various stakeholders and acquire a more comprehensive understanding of the overall acceptability of the intervention. The crossover design is another strength as it allows each patient participant to be their own control, thus reducing confounding factors that are usually present in between-subject designs [50,51]. Moreover, the crossover design will allow for patient participants able to self-report to share their preference between the POMI and the CTL because they will experience both interventions. Information about participant preference will add rich qualitative data beyond the quantitative comparison of preliminary efficacy [41,51]. The crossover design is relevant to use during procedures that are planned within a short period of time (eg, molar extractions at 2 different times) [50]. In addition to intervention comparisons and patient participant preferences, we will be able to determine the pain and distress differences individually (numerically) and compute the proportion of patient participants for whom the treatment was effective in reducing pain by more than 1 point on a 0-10 NRS, for example [51]. The crossover design will also enable us to describe whether any reduction in pain or distress was qualified as meaningful by the patient participant (for those able to self-report). These data could later contribute to the understanding of the minimally clinically significant difference in procedural pain in the adult ICU population [51]. Knowing that patient participants will receive both interventions as part of the crossover design may allow them to think more objectively about pain levels in each intervention period (thus minimizing the placebo effect) compared to if they participated in a parallel-group trial, in which the patient participants’ hope to receive the intervention may influence their pain rating [50]. Finally, blinding patient participants unable to self-report will reduce the risk of bias based on these participants’ expectations of music efficacy to reduce pain.

### Methodological Limitations and Mitigation Strategies

The Initiative on Methods, Measurement, and Pain Assessment in Clinical Trials recommendations state that the use of a crossover trial at an earlier stage study, followed by confirmation of the results in a larger parallel-group study, is an efficient approach, as long as a washout period is carefully planned to minimize or avoid carryover effects [50]. To allow for any pain-reducing effect of the POMI to dissipate before the CTL, there will be a washout period of at least 4 hours between both intervention periods, based on previous data on the duration of the analgesic effect of music on pain [32]. Due to the sample size and to evaluate the time effect, the Friedman and Wilcoxon rank sum tests will be used when analyzing pain-related data for each subgroup [51]. To avoid response shifts due to the crossover design, baseline pain intensity will be measured for each intervention period (T_0_ in Figure 1). Because blinding will be difficult with patient participants receiving both the POMI and the CTL, there is a risk of ascertainment bias for patient participants. To address this, patient participants able to self-report will be asked about their perception of how effective they think the music is compared to the CTL. To minimize possible interruptions and noise during intervention periods, the nurse responsible for the patient will be met to discuss and plan for the best time to start the intervention period, prior to a scheduled bed turn.

Due to the current context of the pandemic and this study being conducted in an ICU setting, it is possible that limited family visits and nursing staff shortages will impact recruitment from these populations. To mitigate these possible limitations, meeting families at the bedside will be coordinated with the nursing staff.

## Abbreviations

bpm: beats per minute
CPOT: Critical-Care Pain Observation Tool
CTL: control intervention
ICU: intensive care unit
NRS: numeric rating scale
POMI: patient-oriented music intervention
RCT: randomized controlled trial
TAP: treatment acceptability and preferences

## Data Availability

The data sets generated during and analyzed during the current study are available from the corresponding author on reasonable request.

## References

[1] Devlin JW, Skrobik Y, Gélinas C, Needham DM, Slooter AJC, Pandharipande PP, Watson PL, Weinhouse GL, Nunnally ME, Rochwerg B, Balas MC, van den Boogaard M, Bosma KJ, Brummel NE, Chanques G, Denehy L, Drouot X, Fraser GL, Harris JE, Joffe AM, Kho ME, Kress JP, Lanphere JA, McKinley S, Neufeld KJ, Pisani MA, Payen J, Pun BT, Puntillo KA, Riker RR, Robinson BRH, Shehabi Y, Szumita PM, Winkelman C, Centofanti JE, Price C, Nikayin S, Misak CJ, Flood PD, Kiedrowski K, Alhazzani W (2018). Clinical practice guidelines for the prevention and management of pain, agitation/sedation, delirium, immobility, and sleep disruption in adult patients in the ICU. Crit Care Med.

[2] Cepeda MS, Carr DB, Lau J, Alvarez H (2006). Music for pain relief. Cochrane Database Syst Rev.

[3] Cole LC, LoBiondo-Wood G (2014). Music as an adjuvant therapy in control of pain and symptoms in hospitalized adults: a systematic review. Pain Manag Nurs.

[4] Lee JH (2016). The effects of music on pain: a meta-analysis. J Music Ther.

[5] Martin-Saavedra JS, Vergara-Mendez LD, Pradilla I, Vélez-van-Meerbeke A, Talero-Gutiérrez C (2018). Standardizing music characteristics for the management of pain: a systematic review and meta-analysis of clinical trials. Complement Ther Med.

[6] Meghani N, Tracy MF, Hadidi NN, Lindquist R (2017). Part I: the effects of music for the symptom management of anxiety, pain, and insomnia in critically ill patients: an integrative review of current literature. Dimens Crit Care Nurs.

[7] Nilsson U (2008). The anxiety- and pain-reducing effects of music interventions: a systematic review. AORN J.

[8] Hole J, Hirsch M, Ball E, Meads C (2015). Music as an aid for postoperative recovery in adults: a systematic review and meta-analysis. Lancet.

[9] Richard-Lalonde M, Gélinas C, Boitor M, Gosselin E, Feeley N, Cossette S, Chlan LL (2020). The effect of music on pain in the adult intensive care unit: a systematic review of randomized controlled trials. J Pain Symptom Manage.

[10] Gosselin É, Richard-Lalonde M (2019). Role of family members in pain management in adult critical care. AACN Adv Crit Care.

[11] Poulsen MJ, Coto J (2018). Nursing music protocol and postoperative pain. Pain Manag Nurs.

[12] Happ MB, Garrett K, Thomas DD, Tate J, George E, Houze M, Radtke J, Sereika S (2011). Nurse-patient communication interactions in the intensive care unit. Am J Crit Care.

[13] Happ MB, Seaman JB, Nilsen ML, Sciulli A, Tate JA, Saul M, Barnato AE (2015). The number of mechanically ventilated ICU patients meeting communication criteria. Heart Lung.

[14] Ames N, Shuford R, Yang L, Moriyama B, Frey M, Wilson F, Sundaramurthi T, Gori D, Mannes A, Ranucci A, Koziol D, Wallen GR (2017). Music listening among postoperative patients in the intensive care unit: a randomized controlled trial with mixed-methods analysis. Integr Med Insights.

[15] Broscious SK (1999). Music: an intervention for pain during chest tube removal after open heart surgery. Am J Crit Care.

[16] Ciğerci Y, Özbayır T (2016). The effects of music therapy on anxiety, pain and the amount of analgesics following coronary artery surgery. Turk Gogus Kalp Dama.

[17] Kyavar M, Karkhaneh S, Rohanifar R, Azarfarin R, Sadeghpour A, Alizadehasl A, Ghadrdoost B (2016). Effect of preferred music listening on pain reduction in mechanically ventilated patients after coronary artery bypass graft surgery. Res Cardiovasc Med.

[18] Chiasson AM, Linda Baldwin A, McLaughlin C, Cook P, Sethi G (2013). The effect of live spontaneous harp music on patients in the intensive care unit. Evid Based Complement Alternat Med.

[19] Jaber S, Bahloul H, Guétin S, Chanques G, Sebbane M, Eledjam J-J (2007). Effects of music therapy in intensive care unit without sedation in weaning patients versus non-ventilated patients. Ann Fr Anesth Reanim.

[20] Sanjuán Naváis M, Via Clavero G, Vázquez Guillamet B, Moreno Duran A, Martínez Estalella G (2013). Effect of music on anxiety and pain in patients with mechanical ventilation. Enferm Intensiva.

[21] Chan MF (2007). Effects of music on patients undergoing a C-clamp procedure after percutaneous coronary interventions: a randomized controlled trial. Heart Lung.

[22] Cooke M, Chaboyer W, Schluter P, Foster M, Harris D, Teakle R (2010). The effect of music on discomfort experienced by intensive care unit patients during turning: a randomized cross-over study. Int J Nurs Pract.

[23] Shultis C (2012). Effects of Music Therapy vs. Music Medicine on Physiological and Psychological Parameters of intensive care patients: A randomized controlled trial.

[24] Guilbaut V (2017). Impact of the standardized musical intervention Music Care © on the painful procedures of critically ill patients [Apport de l'intervention musicale standardisée type Music Care© sur les soins douloureux des patients vigiles en soins critiques].

[25] Bell ML, Whitehead AL, Julious SA (2018). Guidance for using pilot studies to inform the design of intervention trials with continuous outcomes. Clin Epidemiol.

[26] Hertzog MA (2008). Considerations in determining sample size for pilot studies. Res Nurs Health.

[27] Boitor M, Martorella G, Maheu C, Laizner AM, Gélinas C (2018). Effects of massage in reducing the pain and anxiety of the cardiac surgery critically ill-a randomized controlled trial. Pain Med.

[28] Gélinas C, Bérubé M, Puntillo KA, Boitor M, Richard-Lalonde M, Bernard F, Williams V, Joffe AM, Steiner C, Marsh R, Rose L, Dale CM, Tsoller DM, Choinière M, Streiner DL (2021). Validation of the critical-care pain observation tool-neuro in brain-injured adults in the intensive care unit: a prospective cohort study. Crit Care.

[29] Davidson JE, Aslakson RA, Long AC, Puntillo KA, Kross EK, Hart J, Cox CE, Wunsch H, Wickline MA, Nunnally ME, Netzer G, Kentish-Barnes N, Sprung CL, Hartog CS, Coombs M, Gerritsen RT, Hopkins RO, Franck LS, Skrobik Y, Kon AA, Scruth EA, Harvey MA, Lewis-Newby M, White DB, Swoboda SM, Cooke CR, Levy MM, Azoulay E, Curtis JR (2017). Guidelines for family-centered care in the neonatal, pediatric, and adult ICU. Crit Care Med.

[30] Sealed Envelope Ltd.

[31] Spotify AB (2019). Get recommendations based on seeds.

[32] Sen H, Yanarateş O, Sızlan A, Kılıç E, Ozkan S, Dağlı G (2010). The efficiency and duration of the analgesic effects of musical therapy on postoperative pain. Agri.

[33] Sidani S, Epstein DR, Bootzin RR, Moritz P, Miranda J (2009). Assessment of preferences for treatment: validation of a measure. Res Nurs Health.

[34] Streiner DL, Norman GR, Cairney J (2015). Health Measurement Scales: A Practical Guide to Their Development and Use (5 edn).

[35] Epstein DR, Babcock-Parziale JL, Haynes PL, Herb CA (2012). Insomnia treatment acceptability and preferences of male Iraq and Afghanistan combat veterans and their healthcare providers. J Rehabil Res Dev.

[36] Bérubé M, Gélinas C, Martorella G, Feeley N, Côté J, Laflamme G, Rouleau DM, Choinière M (2018). Development and acceptability assessment of a self-management intervention to prevent acute to chronic pain transition after major lower extremity trauma. Pain Manag Nurs.

[37] Martorella G, Gélinas C, Purden M (2014). Acceptability of a web-based and tailored intervention for the self-management of pain after cardiac surgery: the perception of women and men. JMIR Res Protoc.

[38] Sidani S, Brewster J, Miranda J, Walkerly S, Belita E (2016). Exploring the contribution of treatment factors to preferences for smoking cessation interventions. Int Arch Nurs Health Care.

[39] Sidani S, Braden CJ (2011). Design, evaluation, and translation of nursing interventions.

[40] Eldridge SM, Chan CL, Campbell MJ, Bond CM, Hopewell S, Thabane L, Lancaster GA, PAFS consensus group (2016). CONSORT 2010 statement: extension to randomised pilot and feasibility trials. BMJ.

[41] Dwan K, Li T, Altman DG, Elbourne D (2019). CONSORT 2010 statement: extension to randomised crossover trials. BMJ.

[42] Gélinas C, Joffe A, Szumita P, Payen J-F, Bérubé M, Shahiri TS, Boitor M, Chanques G, Puntillo KA (2019). A psychometric analysis update of behavioral pain assessment tools for noncommunicative, critically ill adults. AACN Adv Crit Care.

[43] Gélinas C (2007). The faces pain thermometer: a new tool for critically ill adults. Perspect Infirm.

[44] Puntillo KA, Max A, Timsit J, Ruckly S, Chanques G, Robleda G, Roche-Campo F, Mancebo J, Divatia JV, Soares M, Ionescu DC, Grintescu IM, Maggiore SM, Rusinova K, Owczuk R, Egerod I, Papathanassoglou EDE, Kyranou M, Joynt GM, Burghi G, Freebairn RC, Ho KM, Kaarlola A, Gerritsen RT, Kesecioglu J, Sulaj MMS, Norrenberg M, Benoit DD, Seha MSG, Hennein A, Pereira FJ, Benbenishty JS, Abroug F, Aquilina A, Monte JRC, An Y, Azoulay E (2018). Pain distress: the negative emotion associated with procedures in ICU patients. Intensive Care Med.

[45] Puntillo KA, Neuhaus J, Arai S, Paul SM, Gropper MA, Cohen NH, Miaskowski C (2012). Challenge of assessing symptoms in seriously ill intensive care unit patients: can proxy reporters help?. Crit Care Med.

[46] IBM Corporation (2020). SPSS Statistics for Windows Version 27.

[47] Creswell J, Plano Clark VL, Salmon H (2018). Designing and Conducting Mixed Methods Research (3rd Edition).

[48] Perepletchikova F, Kazdin AE (2005). Treatment integrity and therapeutic change: issues and research recommendations. Clin Psychol: Sci Pract.

[49] Hagermoser SL, Kratochwill T (2009). Toward developing a science of treatment integrity: introduction to the special series. School Psych Rev.

[50] Gewandter JS, Dworkin RH, Turk DC, McDermott MP, Baron R, Gastonguay MR, Gilron I, Katz NP, Mehta C, Raja SN, Senn S, Taylor C, Cowan P, Desjardins P, Dimitrova R, Dionne R, Farrar JT, Hewitt DJ, Iyengar S, Jay GW, Kalso E, Kerns RD, Leff R, Leong M, Petersen KL, Ravina BM, Rauschkolb C, Rice ASC, Rowbotham MC, Sampaio C, Sindrup SH, Stauffer JW, Steigerwald I, Stewart J, Tobias J, Treede R, Wallace M, White RE (2014). Research designs for proof-of-concept chronic pain clinical trials: IMMPACT recommendations. Pain.

[51] Hui D, Zhukovsky DS, Bruera E (2015). Which treatment is better? Ascertaining patient preferences with crossover randomized controlled trials. J Pain Symptom Manage.

